# Semantic annotation in biomedicine: the current landscape

**DOI:** 10.1186/s13326-017-0153-x

**Published:** 2017-09-22

**Authors:** Jelena Jovanović, Ebrahim Bagheri

**Affiliations:** 1 Department of Software Engineering, University of Belgrade, 154 Jove Ilica Street, Belgrade, Serbia; 2 Department of Electrical Engineering, Ryerson University, 245 Church Street, Toronto, Canada

**Keywords:** Natural language processing (NLP), Biomedical ontologies, Semantic technologies, Biomedical text mining, Semantic annotation

## Abstract

The abundance and unstructured nature of biomedical texts, be it clinical or research content, impose significant challenges for the effective and efficient use of information and knowledge stored in such texts. Annotation of biomedical documents with machine intelligible semantics facilitates advanced, semantics-based text management, curation, indexing, and search. This paper focuses on annotation of biomedical entity mentions with concepts from relevant biomedical knowledge bases such as UMLS. As a result, the meaning of those mentions is unambiguously and explicitly defined, and thus made readily available for automated processing. This process is widely known as semantic annotation, and the tools that perform it are known as semantic annotators.

Over the last dozen years, the biomedical research community has invested significant efforts in the development of biomedical semantic annotation technology. Aiming to establish grounds for further developments in this area, we review a selected set of state of the art biomedical semantic annotators, focusing particularly on general purpose annotators, that is, semantic annotation tools that can be customized to work with texts from any area of biomedicine. We also examine potential directions for further improvements of today’s annotators which could make them even more capable of meeting the needs of real-world applications. To motivate and encourage further developments in this area, along the suggested and/or related directions, we review existing and potential practical applications and benefits of semantic annotators.

## Background

Over the last few decades, huge volume of digital unstructured textual content have been generated in biomedical research and practice, including a range of content types such as scientific papers, medical reports, and physician notes. This has resulted in massive and continuously growing collections of textual content that need to be organized, curated and managed in order to be effectively used for both clinical and research purposes. Clearly, manual curation and management of such “big” corpora are infeasible, and hence, the biomedical community has long been examining and making use of various kinds of Natural Language Processing (NLP) methods and techniques to, at least partially, facilitate their use.

In this paper, we focus on a specific NLP task, namely the extraction and disambiguation of entities mentioned in biomedical textual content. Early efforts in biomedical information extraction were devoted to Named Entity Recognition (NER), the task of recognizing specific types of biomedical entities mentioned in text [1]. For instance, in the sentence “The patient was diagnosed with upper respiratory tract infection”, a NER tool would recognize that the phrase “respiratory tract infection” denotes a disease, but would not be able to determine what particular disease it is. Semantic annotation, the NLP task of interest to this paper, makes a significant advance, by not only recognizing the type of an entity, but also uniquely linking it to its appropriate corresponding entry in a well-established knowledge base. In the given example, a semantic annotator would not only recognize that the phrase “respiratory tract infection” represents a disease, but would also identify what disease it is by connecting the phrase with the concept C0035243 denoting ‘Respiratory Tract Infections’ from the UMLS Metathesaurus (see Table 1). This way, the semantics of biomedical texts is made accessible to software programs so that they can facilitate various laborious and time consuming tasks such as search, classification, or organization of biomedical content.

**Table 1:** An overview of ontologies, thesauri and knowledge bases used by biomedical semantic annotation tools discussed in the paper

BioPortal (http://bioportal.bioontology.org/)	A major repository of biomedical ontologies, currently hosting over 500 ontologies, controlled vocabularies and terminologies. Its Resource Index provides an ontology-based unified index of and access to multiple heterogeneous biomedical resources (annotated with BioPortal ontologies).
DBpedia (http://wiki.dbpedia.org/)	“Wikipedia for machines”, that is, a huge KB developed through a community effort of extracting information from Wikipedia and representing it in a structured format suitable for automated machine processing. It is the central hub of the Linked Open Data Cloud.
LLD - Linked Life Data (https://datahub.io/dataset/linked-life-data/)	LLD platform provides access to a huge KB that includes and semantically interlinks knowledge about genes, proteins, molecular interactions, pathways, drugs, diseases, clinical trials and other related types of biomedical entities. It is part of the Linked Open Data Cloud (http://lod-cloud.net/)
NCBI Biosystems Database (https://www.ncbi.nlm.nih.gov/biosystems)	Repository providing integrated access to structured data and knowledge about biological systems and their components: genes, proteins, and small molecules. The NCBI Taxonomy contains the names and phylogenetic lineages of all the organisms that have molecular data in the NCBI databases.
OBO - Open Biomedical Ontologies (http://www.obofoundry.org/)	Community of ontology developers devoted to the development of a family of interoperable and scientifically accurate biomedical ontologies. Well known OBO ontologies include: • *Chemical Entities of Biological Interest (ChEBI*) - focused on molecular entities, molecular parts, atoms, subatomic particles, and biochemical roles and applications • *Gene Ontology (GO)* - aims to standardize the representation of gene and gene product attributes; consists of 3 distinct sub-ontologies: Molecular Function, Biological Process, and Cellular Component • *Protein Ontology (PRO)* - provides a structural representation of protein-related entities
SNOMED CT (http://www.ihtsdo.org/snomed-ct)	SNOMED CT is considered the world’s most comprehensive and precise, multilingual health terminology. It is used for the electronic exchange of clinical health information. It consists of concepts, concept descriptions (i.e., several terms that are used to refer to the concept), and concept relationships.
UMLS (Unified Medical Language System) Metathesaurus (https://www.nlm.nih.gov/research/umls/knowledge_sources/metathesaurus/)	The most well-known and widely used knowledge source in the biomedical domain. It assigns a unique identifier (CUI) to each medical concept and connects concepts to each other thus forming a graph-like structure; each concept (i.e. CUI) is associated with its ‘semantic type’, a broad category such as Gene, Disease or Syndrome; each concept is also associated with several terms used to refer to that concept in biomedical texts; these terms are pulled from nearly 200 biomedical vocabularies. Some well-known vocabularies that have been used by biomedical semantic annotators include: • *Human Phenotype Ontology* (*HPO*) contains terms that describe phenotypic abnormalities encountered in human disease, and is used for large-scale computational analysis of the human phenome. • *Logical Observation Identifiers Names and Codes* (*LOINC*) provides standardized vocabulary for laboratory and other clinical observations, and is used for exchange and/or integration of clinical results from several disparate sources. • *Medical Subject Headings* (*MeSH*) is a controlled vocabulary thesaurus created and maintained by U.S. National Library of Medicine (NLM), and has been primarily used for indexing articles in PubMed • *RxNorm* provides normalized names for clinical drugs and links between many of the drug vocabularies commonly used in pharmacy management and drug interaction software.
UniProtKb/Swiss-Prot (http://www.uniprot.org/uniprot/)	Part of UniProtKB, a comprehensive protein sequence KB, which contains manually annotated entries. The entries are curated by biologists, regularly updated and cross-linked to numerous external databases, with the ultimate objective of providing all known relevant information about a particular protein.

While a suite of biomedical semantic annotation tools is available for practical use, the biomedical community is yet to heavily engage in and leverage the benefits of such tools. The goal of this paper is to introduce (i) some of the benefits and application use cases of biomedical semantic annotation technology, (ii) a selection of the publicly available general purpose semantic annotation tools for the biomedical domain, i.e., semantic annotators that are not specialized for a particular biomedical entity type, but can detect and normalize entities of multiple types in one pass, and (iii) potential areas where the work in the biomedical semantic annotation domain can be strengthened or expanded. While the overview of application cases and state of the art tools can be of relevance to practitioners in the biomedical domain, with the summary of potential areas for further research, we are also targeting researchers who are familiar with NLP, semantic technologies, and semantic annotation in general, but have not been dealing with the biomedical domain, as well as those who are well aware of biomedical semantic technologies, but have not been working on semantic annotation. By providing researchers with an insight into the current state of the art in biomedical semantic annotation in terms of the approaches and tools, as well as the research challenges, we aim to offer them a basis for engagement with semantic annotation technology within the biomedical domain and thus support even further developments in the field.

The following section provides several examples of practical benefits achievable through semantic annotation of biomedical texts (see also Table 2). The paper then examines the available tool support, focusing primarily on general purpose biomedical annotators (Tables 3 and 4). Still, considering the relevance and large presence of entity-specific biomedical annotators, i.e., tools developed specifically for semantic annotation of a particular type of biomedical entities such as genes or chemicals, we provide an overview of these tools, as well. While examining the available tool support, we also consider biomedical knowledge resources required for semantic annotation (Table 1), as well as resources used for evaluating the tools’ performance (Table 5). This is followed by a discussion of the challenges that are preventing current semantic annotators from achieving their full potential.

**Table 2:** Example application cases of biomedical semantic annotation tools

Application Case (AC)	The role of semantic annotation tool in the AC	Biomedical resources relevant for the AC (or representative examples, if multiple)
Semantic search of biomedical tools and services [6]	Sematic search of biomedical tools and services enabled by semantic annotation of users’ (free-form) queries with concepts from UMLS Metathesaurus	Catalogs of and social spaces created around biomedical tools and services, e.g.: - myExperiment (http://www.myexperiment.org/) - BioCatalogue (https://www.biocatalogue.org/)
Semantic search of domain specific scientific literature [74]	Semantic annotation of PubMed entries with ontological concepts related to genes and proteins	Ontologies used for the annotation of biomedical references (PubMed entries): - Gene Ontology - GO (http://geneontology.org/) - Universal Protein Resource - UniProt (http://www.uniprot.org/uniprot/)
Improved clinical decision making [75]	Extraction of key clinical concepts (UMLS-based) required for supporting clinical decision making; the concepts are extracted from biomedical literature and clinical text sources	Sources of biomedical texts used to support decision making: - PubMed Central (PMC) Open Access Subset (https://www.ncbi.nlm.nih.gov/pmc/tools/openftlist/) - MEDLINE abstracts (https://www.nlm.nih.gov/bsd/pmresources.html)
Unambiguous description of abbreviations [10]	Extended (long) forms of abbreviations are matched against both UMLS and DBpedia concepts, thus not only disambiguating the long forms, but also connecting UMLS and DBpedia KBs	Allie - a search service for abbreviations and their long forms (http://allie.dbcls.jp/)

**Table 3:** General purpose biomedical semantic annotation tools (Part I)

	cTAKES [4]	NOBLE Coder [20]	MetaMap [31, 32]	NCBO annotator [14]
Modularity/configuration options	Modular text processing pipeline	Vocabulary (terminology); Term matching options and strategies	Text processing pipeline; Vocabulary (terminology); Term matching options and strategies	Vocabulary (terminology); Term matching options
Disambiguation of terms	Enabled through integration of the YTEX component [8]	Instead of through WSD, it uses heuristics to choose one concept among candidate concepts for the same piece of input text	Supported; based on: - removal of word senses based on a manual study of UMLS ambiguity - a WSD algorithm that chooses a concept with the most likely semantic type for a given context	Not supported
Vocabulary (terminology)	Subset of UMLS, namely SNOMED CT and RxNORM	Several pre-built vocabularies, based on subsets of UMLS (e.g. SNOMED CT, MeSH, RxNORM)	UMLS Metathesaurus	UMLS Metathesaurus and BioPortal ontologies (over 330 ontologies)
Speed*	Suitable for real-time processing	Suitable for real-time processing	Better for off-line batch processing	Suitable for real-time processing
Implementation form	Software (Java) library; Stand-alone application	Software (Java) library; Stand-alone application	Software library; originally version in Prolog; Java implementation, known as MMTX, is also available	RESTful Web service
Availability	open source; available under Apache License, v.2.0	open-source; available under GNU Lesser General Public License v3	open source; terms and conditions at: https://metamap.nlm.nih.gov/MMTnCs.shtml	closed source, but freely available
Specific features	Better performance on clinical texts than on biomedical scientific literature (its NLP components are trained on clinical texts)	Offers user interface for creating custom terminologies (to be used for annotation) by selecting and merging elements from several different thesauri/ontologies	Primarily developed for annotation of biomedical literature (MEDLINE/PubMed citations); performs better on this kind of text than clinical notes	It uses MGrep term-to-concept matching tool to get primary set of annotations; these are then extended using different forms of ontology-based semantic matching
URL	http://ctakes.apache.org/	http://noble-tools.dbmi.pitt.edu/	https://metamap.nlm.nih.gov/	https://bioportal.bioontology.org/annotator

*Note that speed estimates are based on the experimental results reported in the literature; those experiments were done with corpora of up to 200 documents (paper abstracts or clinical notes); the given estimates might not hold for significantly larger corpora

**Table 4:** General purpose biomedical semantic annotation tools (Part II)

	BeCAS [36]	Whatizit [38]	ConceptMapper [21]	Neji [40]
Modularity/configuration options	Semantic types (i.e. types of entities to annotate)	pre-built pipelines for several biomedical types (see Specific features)	Text processing pipeline; Term matching options and strategies	Modular text processing pipeline
Disambiguation of terms	No information available	Not supported	Not supported	Instead of through WSD, it uses a set of heuristics rules to identify and remove annotations of lower importance
Vocabulary (terminology)	Custom built vocabulary by using concepts from multiple sources, such as UMLS, NCBI BioSystems, ChEBI, and the Gene Ontology.	The use of the vocabulary depends on the type of entity a pipeline is specialized for (e.g. NCBI KB for species, or Gene Ontology for genes)	General purpose dictionary lookup tool, not tied to any vocabulary	Not tied to any particular vocabulary
Speed^a^	Suitable for real-time processing	Suitable for real-time processing	Suitable for real-time processing	Suitable for real-time processing
Implementation form	Software (Python) library; RESTful Web service; Javascript widget	SOAP Web service	Software (Java) library; part of the UIMA NLP framework [28]	RESTful Web service
Availability	open source; available under Attribution-NonCommercial 3.0 Unported license	closed source, but freely available	open source; available under Apache License, v.2.0	open source; available under Attribution-NonCommercial 3.0 Unported license
Specific features	Primarily aimed for annotation of biomedical research papers; focused on annotation of several (11) types of biomedical entities, including species, microRNAs, enzymes, chemicals, drugs, diseases, etc.	Offers several pre-built pipelines for specific entity types; e.g. whatizitGO identifies proteins based on the Gene Ontology (GO), while whatizitChemical annotates chemical entities based on ChEBI	Not specifically developed for the biomedical domain, but is a general purpose dictionary lookup tool	Includes modules for both ML and dictionary-based annotation; can automatically combine annotations generated by different modules
URL	http://bioinformatics.ua.pt/becas/	http://www.ebi.ac.uk/webservices/whatizit	https://uima.apache.org/sandbox.html#concept.mapper.annotator	https://github.com/BMDSoftware/neji

^a^Note that speed estimates are based on the experimental results reported in the literature; those experiments were done with corpora of up to 200 documents (paper abstracts or clinical notes); the given estimates might not hold for significantly larger corpora

**Table 5:** Corpora used for evaluation of biomedical semantic annotators. The table includes corpora that were used in the reported use cases (“Benefits and Use Cases” section, Table 2), and/or benchmarking of the discussed tools ("Summary of benchmarking results" and "Entity-specific biomedical annotation tools" sections)

AnEM - Anatomical Entity Mention [76]	The corpus consists of 500 documents selected randomly from citation abstracts and full-text biomedical research papers (from PubMed); it is manually annotated (over 3000 annotations) with anatomical entities. The corpus is available under the open CC-BY-SA license. URL: http://www.nactem.ac.uk/anatomy/
BC4GO [77]	The corpus, developed for the BioCreative IV shared task, consists of 200 articles (over 5000 text passages) from Model Organism Databases; these articles were manually annotated with more than 1356 distinct GO terms. In addition to the core elements of GO annotations - a gene or gene product, a GO term, and a GO evidence code - the corpus also includes the GO evidence text. URL: http://www.biocreative.org/tasks/biocreative-iv/track-4-GO/
CALBC - Collaborative Annotation of a Large Biomedical Corpus [78]	A very large, publicly shared corpus of Medline abstracts automatically annotated with biomedical entities; the small corpus comprises ~175 K abstracts, whereas the big one consists of more than 714 K abstracts; since annotations were not made by humans but several annotation systems (and then aggregated), it is referred to as “silver standard”. URL: http://www.ebi.ac.uk/Rebholz-srv/CALBC/corpora/resources.html
Chemical Disease Relation (CDR) [79]	The corpus, developed for the BioCreative V shared task, consists of 1500 PubMed articles with 4409 annotated chemicals, 5818 diseases, and 3116 chemical-disease interactions. MeSH is used as the controlled vocabulary. As BC4GO, this corpus is available exclusively for scientific, educational, and/or non-commercial purposes. URL: http://www.biocreative.org/tasks/biocreative-v/track-3-cdr/
CRAFT - the Colorado Richly Annotated Full Text corpus [80]	Publicly available, human annotated (gold standard) corpus of full-text biomedical journal articles; it consists of 67 document and 87,674 human annotations URL: http://bionlp-corpora.sourceforge.net/CRAFT/
GENETAG [81]	Publicly available corpus of 20 K Medline sentences manually annotated with gene/protein names. Part of the corpus (15 K sentences) was used for the BioCreative I challenge (Gene Mention Identification task), and the rest (5 K sentences) was used as test data for BioCreative II competition (Gene Mention Tagging Task). URL: https://github.com/openbiocorpora/genetag An updated version of this corpus, named GENETAG-05, is part of a broader MedTag annotated corpus that was used in the BioCreative I challenge; it is available at: ftp://ftp.ncbi.nlm.nih.gov/pub/lsmith/MedTag/
GENIA [82]	Open access manually annotated corpora consisting of 2000 Medline abstracts (400,000+ words) with almost 100,000 annotations for biological terms. Terms are annotated with concepts from the GENIA ontology, a formal model of cell signaling reactions in humans (the ontology is provided together with the corpus). Available from the following repository: http://corpora.informatik.hu-berlin.de/
2010 i2b2/VA corpus [83]	The corpus consists of manually annotated de-identified clinical records (discharge summaries and progress reports) from three medical centers. It was originally created for the 2010 i2b2/VA NLP challenge to support 3 kinds of tasks: extraction of medical concepts from patient reports; assigning assertion types to medical problem concepts; and determining the type of relation between medical problems, tests, and treatments. The corpus consists of 394 annotated training reports, 477 annotated test reports, and 877 unannotated reports. The corpus is made available to the research community from https://i2b2.org/NLP/DataSets under data use agreements.
JNLPBA [84]	A publicly available manually annotated corpus originally created for the Bio-Entity Recognition Task at BioNLP/NLPBA 2004. The training set consists of 2000 Medline abstracts extracted from the GENIA Version 3.02 corpus; the data set is annotated with five entity types: Protein, DNA, RNA, Cell_line, and Cell_type. The test set consists of 404 annotated Medline abstracts, also from the GENIA project; a half of this data set is from the same domain as that of the training data, whereas the other half is from the super domain of blood cells and transcription factors. URL: http://www.geniaproject.org/shared-tasks/bionlp-jnlpba-shared-task-2004
NCBI Disease corpus [85]	Publicly available, manually annotated corpus of 793 PubMed abstracts; 6892 disease mentions are annotated with concepts from Medical Subject Headings (MeSH) and Online Mendelian Inheritance in Man (OMIM) vocabularies. URL: https://www.ncbi.nlm.nih.gov/CBBresearch/Dogan/DISEASE/
Mantra Gold Standard Corpus [73]	Publicly available multilingual gold-standard corpus for biomedical concept recognition. It includes text from different types of parallel corpora (Medline abstract titles, drug labels, biomedical patent claims) in English, French, German, Spanish, and Dutch. It contains 5530 annotations based on a subset of UMLS that covers a wide range of semantic groups. URL: http://biosemantics.org/index.php/resources/mantra-gsc
ShARe - Shared Annotated Resources [86]	Gold standard corpus of de-identified clinical free-text notes; it includes 199 documents and 4211 human annotations; originally prepared for the ShARe/CLEF eHealth Evaluation Lab focused on NLP and information retrieval tasks for clinical care. URL: https://sites.google.com/site/shareclefehealth/data

## Benefits and use cases

### Better use of electronic medical record (EMR) in clinical practice

Electronic medical records (EMRs) are considered valuable source of clinical information, ensuring effective and reliable information exchange among physicians and departments participating in patient care, and supporting clinical decision making. However, EMRs largely consist of unstructured, free-form textual content that require manual curation and analysis performed by domain experts. A recent study examining the allocation of physician time in ambulatory practice [2] confirmed the findings of previous similar studies (e.g. [3]), namely that physicians spend almost twice as much time on the management of EMRs and related desk work than on direct clinical face time with patients. Considering the inefficiency of manual curation of EMRs, automation of the process is required if the potentials of EMRs are to be exploited in clinical practice [4].

Semantic annotators provide the grounds for the required automation by extracting clinical terms from free-form text of EMRs, and disambiguating the extracted terms with concepts of a structured vocabulary, such as UMLS Metathesaurus. The identified concepts can be subsequently used to search a repository of biomedical literature or evidence-based clinical resources, in order to enrich EMRs with information pertinent to the patient’s state. The extracted terms can also be used for making summaries of clinical notes and articles [5].

### Improved search and retrieval of resources for biomedical research

Publicly available biomedical data, tools, services, models and computational workflows continuously increase in number, size and complexity. While this ever-growing abundance of valuable resources opens up unprecedented opportunities for biomedical research, it is also making it ever more challenging for researchers to efficiently discover and use the resources required for accomplishing their tasks [6]. Hence, automation of the search and discovery processes has turned into a necessity.

Clinical information stored in EMRs is also important in medical research, e.g., for comparative effectiveness research, and epidemiological and clinical research studies [7]. Considering the unstructured nature of EMRs and their abundance, automated document classification and information extraction methods are essential for assuring the efficiency and effectiveness of search and retrieval of relevant information from large EMR collections. Semantic annotation techniques can play a significant role in this regard. For instance, they can be used to extract domain-specific concepts that could serve as discriminative features for building automated classifiers of clinical documents [8]. Based on such classification, clinical documents can be searched more effectively [7]. Furthermore, semantic concepts extracted from biomedical literature can also be used for semantic indexing and retrieval of biomedical publications [9] or biomedical tools and services [6]. In particular, biomedical Information Retrieval systems use semantic annotators to expand the users’ queries with concepts and terms from vocabularies/ontologies (mapping the query text to the appropriate ontology concepts, and then expanding the query with the terms associated with the mapped concepts), as well as to classify the retrieved documents based on their content or the occurrence of specific topics in the documents [1].

### Disambiguation of abbreviations

Polysemous abbreviations are frequently present in biomedical literature and clinical texts making it difficult for researchers and clinical practitioners to understand texts that are outside the strict area of their expertise [10]. According to Chang et al. [11], in biomedical journal articles, abbreviations with six or less characters have on average 4.61 possible meanings. For instance, “ANA” has numerous possible meanings among which the most frequent ones are “antinuclear antibodies”, “American Nurses Association”, “Alko Non-Alcohol”, and “anandamide”.

Semantic annotators combined with general-purpose, machine-readable knowledge bases, such as DBpedia (Table 1), can be used to disambiguate polysemous abbreviations and unambiguously describe abbreviated terms based on the context in which they appear [10]. This can help researchers and practitioners better understand the meaning of such abbreviations.

### Seamless integration of data from disparate sources

Biomedical data are stored and maintained in disparate repositories. For instance, according to the 2016 Molecular Biology Database Update, there are 1685 biological databases [12]. This complicates the tasks of data management, retrieval and exploitation since one needs to, first, locate the repositories that contain the required data; then, to familiarize oneself with the meaning of the attributes and data types used in each repository; and, finally, learn how to access and query the repositories [13].

For this reason, data integration can be highly useful for medical researchers. Jonquet et al. [14] have nicely illustrated the need for seamless integration of data from various medical sources: “a researcher studying the allelic variations in a gene would want to know all the pathways that are affected by that gene, the drugs whose effects could be modulated by the allelic variations in the gene, and any disease that could be caused by the gene, and the clinical trials that have studied drugs or diseases related to that gene. The knowledge needed to address such questions is available in public biomedical resources; the problem is finding [and connecting] that information.”

Ontologies that are used to semantically annotate items in biomedical repositories allow for weaving semantic links both within and across repositories thus establishing a semantic network of biomedical items [13]. If both ontologies and resources they connect (through semantic annotations) are in public domain, the resulting network takes the form of Linked Open Data, as has already been shown in the Linked Life Data initiative (Table 1).

Table 2 provides a more structured view of some application cases for biomedical semantic annotation technology.

## Annotation process, tools and resources

Biomedical texts have several characteristics that make them particularly challenging not only for semantic annotation, but for any NLP task [6]. Some of these characteristics include:

i) Clinical text produced by practitioners often do *not fully adhere to correct grammar, syntactic or spelling rules*, as the following triage note illustrates: “SORE THROAT pt c/o sore throat x 1 week N pt states took antibiotic x 5 days after initiation of sore throat and sx resolved and now back after completed antibiotics N pt tolerating po fluids yet c/o pain on swallowing”;
ii) Biomedical terms are *often polysemous* and thus *prone to ambiguity*; for example, an analysis of over 409 K Medline abstracts revealed that 11.7% of the phrases were ambiguous relative to the UMLS Metathesaurus [15].
iii) These textual corpora frequently use *abbreviations and acronyms that tend to be polysemous* (see Disambiguation of abbreviations section). In addition, clinical texts often contain *non-standard shorthand phrases*, laboratory results and notes on patients’ vital signs, which are often filled with periods and thus can complicate typically straightforward text processing tasks such as sentence splitting [16].
iv) Biomedical texts about or related to gene and protein mentions are particularly challenging for semantic annotation. This is because *every protein* (e.g., SBP2), *has an associated gene, often with the same name* [17]. Furthermore, *multiple genes share symbols and names* (e.g. ‘CAT’ is the name of different genes in several species, namely in cow, chicken, fly, human, mouse, pig, deer and sheep [18]).

To address these and other challenges of unstructured biomedical text, state-of-the-art semantic annotators often rely on a combined use of text processing, large-scale knowledge bases, semantic similarity measures and machine learning techniques [19]. In particular, in the biomedical domain, semantic annotation is typically based on one of the following two general approaches [20]: term-to-concept matching approach and approach based on machine learning (ML) methods.

The term-to-concept matching approach, also referred to as dictionary lookup, is based on matching specific segments of text to a structured vocabulary/dictionary or knowledge base (e.g. UMLS or some of the OBO ontologies, Table 1). The drawback of some of the annotators that implement this approach, e.g., NCBO Annotator [14] and ConceptMapper [21], is the lack of disambiguation ability, meaning that the terms recognized in texts are connected with several possible meanings, i.e., dictionary entries/concepts, instead of being associated with a single meaning that is most appropriate for the given context. For example, in the absence of disambiguation, in the sentence “In patients with DMD, the infiltration of skeletal muscle by immune cells aggravates disease”, the term DMD would be associated with several possible meanings, including Duchenne muscular dystrophy, dystrophin, and DMD gene, whereas only the first one is correct for this given context.

The approaches based on ML methods are often found in annotators developed for specific, well-defined application areas such as annotating drugs in medical discharge summaries [22] or recognizing gene mentions in biomedical papers [23]. These annotators unambiguously detect domain-specific concepts in text, and are typically highly performant on the specific tasks they were developed for. However, as they are often based on supervised ML methods, their development, namely, training of a ML model, requires large expert annotated corpora, which are very expensive to develop. Another drawback of such annotators is that they are only able to recognize specific categories of entities they are trained for, such as genes or diseases, and cannot be applied to recognize concepts from broader vocabularies [24]. The high costs associated with these approaches has led to a shift towards unsupervised or semi-supervised ML methods that require few or no manually labelled data [25]. Furthermore, several recent approaches have considered the idea of *distant supervision* to generate ‘noisy’ labeled data for entity recognition [26] and entity typing [27].

### Semantic biomedical annotation tools

A large number of semantic annotation tools have been developed for the biomedical domain [20, 24]. Many of them have resulted from research projects. Our focus in this paper is on a subset of these tools that have the following characteristics:

- Semantic annotators that have been applied in practice or at least in research projects other than those they originated from. In other words, we are not considering research prototypes, but semantic annotators that have evolved from a research prototype and have demonstrated their robustness for practical use.
- Semantic annotation tools that are available either as software libraries, web services or web applications.
- General-purpose biomedical annotators, i.e., those semantic annotators that are not tied to any particular biomedical task or entity type, but can be configured to work with texts from different biomedical subdomains. This capacity originates from the fact that they are either fully or at least partially grounded in the term-to-concept annotation approach, which is flexible with respect to the annotation terminology.

Tables 3 and 4 gives an overview of the semantic annotation tools that fulfilled the above given criteria and thus were selected for inclusion in our study.^1^ The table compares the selected tools with respect to several characteristic, including those related to the underlying annotation method (configurability and disambiguation), the vocabulary (terminology) the tool relies on, the tool’s speed,^2^ its implementation aspects, and availability. The table also points to some of the tools’ specific features, which are further examined in the tool descriptions given below.

As shown in Tables 3 and 4 and further discussed below, all the tools are configurable in several and often different ways, making it very difficult, if possible at all, to give a fair general comparison of the tools. In other words, we believe that the only way to properly compare these (and similar) annotation tools is in the context of a specific application case, where each tool would be configured based on the application requirements. We expand on this in “Application-specific tool benchmarking” section where we discuss the need for a benchmarking toolkit that would facilitate this kind of application-specific tool benchmarking. Still, to offer some general insight into the annotation capabilities of the selected tools, in “Summary of benchmarking results” section we briefly report on the benchmarking studies that included several of the examined semantic annotators. In the following, we introduce the selected semantic annotation tools and discuss their significant features. The tools are presented in the order that corresponds to their order in Tables 3 and 4.

***Clinical Text Analysis and Knowledge Extraction System (cTAKES)*** [4] is a well-known toolkit for semantic annotation of biomedical documents in general, and clinical research texts in particular. It is built on top of two well-established and widely used open-source NLP frameworks: Unstructured Information Management Architecture - UIMA [28] and OpenNLP [29]. cTAKES is developed in a modular manner, as a pipeline consisting of several text processing components that rely on either rule-based or ML techniques. Recognition of concept mentions and annotation with the corresponding concept identifiers is done by a component that implements a dictionary look-up algorithm. For building the dictionary, cTAKES relies on UMLS. The concept recognition component does not resolve ambiguities that result from identifying multiple concepts for the same text span. Disambiguation is enabled through the integration of YTEX [7] in the cTAKES framework and its pipelines. YTEX is a knowledge-based word sense disambiguation component that relies on the knowledge encoded in UMLS. In particular, YTEX implements an adaptation of the Lesk method [30], which scores candidate concepts for an ambiguous term by summing the semantic relatedness between each candidate concept and the concepts in its context window.

***NOBLE Coder*** [20] is another open-source, general-purpose biomedical annotator. It can be configured to work with arbitrary vocabularies. Besides enabling users to annotate documents with existing vocabularies (terminologies), NOBLE Coder also provides them with a Graphical User Interface where they can create custom terminologies by selecting one or more branches from a set of existing vocabularies, and/or filtering vocabularies by semantic types. It also allows for the dynamic change of the terminology (adding new concepts, removing existing ones) while processing. The flexibility of this annotator also lies in the variety of supported concept matching strategies, aimed at meeting the needs of different kinds of NLP tasks. For example, the ‘best match’ strategy aims at high precision, and thus returns few candidates (at most); as such, it is suitable for concept coding and information extraction NLP tasks. The supported matching strategies allow for annotation of terms consisting of single words, multiple words, and abbreviations. Thanks to its greedy algorithm, NOBLE Coder can efficiently process large textual corpora. To disambiguate terms with more than one associated concept, this tool relies on a set of simple heuristic rules such as giving preference to candidates that map to a larger number of source vocabularies, or candidates where the term is matched in its ‘original’ form, i.e., without being stemmed or lemmatized.

***MetaMap*** [31] is probably the most well-known and most widely used biomedical annotator. It was developed by the U.S. National Library of Medicine. It maps biomedical entity mentions of the input text to the corresponding concepts in the UMLS Metathesaurus. Each annotation includes a score that reflects how well the concept matches the biomedical term/phrase from the input text. The annotation process can be adapted in several ways by configuring various elements of the annotation process such as the vocabulary used, the syntactic filters applied to the input text, and the matching between text and concepts, to name a few. Besides the flexibility enabled by these configuration options, another strong aspect of MetaMap is its thorough and linguistically principled approach to the lexical and syntactic analyses of input text. However, this thoroughness is also the cause of one of MetaMap’s main weaknesses, namely its long processing time, and thus its inadequacy for annotating large corpora. Another weakness lies in its disambiguation approach which is not able to effectively deal with ambiguous terms [32]. In particular, for disambiguation of terms, MetaMap combines two approaches: i) removal of word senses deemed problematic for (literature-centric) NLP usage, based on a manual study of UMLS ambiguity, and ii) a word sense disambiguation algorithm that chooses a concept with the most likely semantic type for a given context [33].

***NCBO annotator*** [14] is provided by the U.S. National Center for Biomedical Ontology (NCBO) as a freely available Web service. It is based on a two-stage annotation process. The first stage relies on a concept recognition tool that uses a dictionary to identify mentions of biomedical concepts in the input text. In particular, NCBO annotator makes use of the MGrep tool [34], which was chosen over MetaMap due to its better performance along several examined dimensions [35]. The dictionary for this annotation stage is built by pulling concept names and descriptions from biomedical ontologies and/or thesauri relevant for the domain of the corpus to be annotated (typically UMLS Metathesaurus and BioPortal ontologies, Table 1). In the second stage, the initial set of concepts, referred to as direct annotations, is extended using the structure and semantics of relevant biomedical ontologies. For instance, semantic distance measures are used to extend the direct annotations with semantically related concepts; the computation of semantic distance is configurable, and can be based, for instance, on the distance between the concepts in the ontology graph. Semantic relations between concepts from different ontologies, established through ontology mappings, serve as another source for finding semantically related concepts that can be used to extend the scope of direct annotations. The NCBO annotator is unique in its approach to associate concept mentions with multiple concepts, instead of finding one concept that would be the best match for the given context.

***BioMedical Concept Annotation System*** **(** ***BeCAS*** **)** [36] is a Web-based tool for semantic annotation of biomedical texts, primarily biomedical research papers. Besides being available through a Web-based user interface, it can be programmatically accessed through a Web-based (RESTful) Application Programing Interface (API), and a widget, easily embeddable in Web pages. Like majority of the aforementioned annotation tools, BeCAS is an open-source modular system, comprising of several modules for text preprocessing including, e.g., sentence splitting, tokenization, lemmatization, among others, as well as modules for concept detection and abbreviation resolution. Most of the concept detection modules in BeCAS apply a term-to-concept matching approach to identify and annotate mentions of several types of biomedical entities, including species, enzymes, chemicals, drugs, diseases, etc. This approach relies on a custom dictionary, i.e., a database of concepts and associated terms, compiled by pulling concepts from various meta-thesauri and ontologies such as UMLS Metathesaurus, NCBI BioSystems database, ChEBI, and the Gene Ontology (Table 1). For the identification of gene and protein mentions and their disambiguation with appropriate concepts, BeCAS makes use of Gimli, an open source tool that implements Conditional Random Fields (CRF) for named entity recognition in biomedical texts [37] (see Entity-specific biomedical annotation tools section).

***Whatizit*** is a freely available Web service for annotation of biomedical texts with concepts from several ontologies and structured vocabularies [38]. Like previously described tools, it is also developed in a modular way so that different components can be combined into custom annotation pipelines, depending on the main theme of the text being processed. For example, *whatizitGO* is a pipeline for identifying Gene Ontology (GO) concepts in the input text, while *whatizitOrganism* identifies species defined in the NCBI taxonomy. In Whatizit*,* concept names are transformed into regular expressions to account for morphological variability in the input texts [39]. Such regular expressions are then compiled into Finite State Automata, which assure quick processing regardless of the length of the text and the size of the used vocabulary; therefore, processing time is linear with respect to the length of the text. Whatizit also offers pipelines that allow for the recognition of biomedical entities of a specific type based on two or more knowledge sources. For instance, *whatizitSwissprotGo* is the pipeline for the annotation of protein mentions based on the UniProtKb/Swiss-Prot knowledge base (Table 1) and the Gene Ontology. Finally, there are more complex pipelines that combine simpler pipelines to enable detection and annotation of two or more types of biomedical entities. For instance, *whatizitEbiMed* incorporates *whatizitSwissprotGo*, *whatizitDrug* and *whatizitOrganism* to allow for the detection and annotation of proteins, drugs and species.

***ConceptMapper*** [21] is a general purpose dictionary lookup tool, developed as a component of the open-source UIMA NLP framework. Unlike the other annotators that have been examined so far, ConceptMapper is the only one that was not specifically developed for the biomedical domain, but is rather generic and configurable-enough to be applicable to any domain. Its flexibility primarily stems from the variety of options for configuring its algorithm for mapping dictionary entries onto input text. For instance, it can be configured to detect entity mentions even when they appear in the text as disjoint multi-word phrases, e.g., in the text “intraductal and invasive mammary carcinoma”, it would recognize “intraductal carcinoma” and “invasive carcinoma” as diagnosis. It can also deal with a variety of ways a concept can be mentioned in the input text, e.g., synonyms and different word forms. This is enabled by a dictionary that for each entry stores several possible variants, and connects them to the same concept. For instance, the entry with the main (canonical) form “spine” would also include variants such as “spinal”, “spinal column”, “vertebral column”, “backbone”, and others, and associates them all with the semantic type AnatomicalSite. Even though ConceptMapper is not originally targeted at the biomedical domain, if properly configured, it can even outperform state-of-the-art biomedical annotators [24]. However, the task of determining the optimal configuration and developing a custom dictionary might be overwhelming for regular users; we return to this topic in “Adaptation to new document type(s) and/or terminologies specific to particular biomedical subdomain” section.

***Neji*** [40] is yet another open source and freely available software framework for annotation of biomedical texts. Its high modularity is achieved by having each text processing task wrapped in an independent module. These modules can be combined in different ways to form different kinds of text processing and annotation pipelines, depending on the requirements of specific annotation tasks. The distinct feature of Neji is its capacity for multi-threaded data processing, which assures high speed of the annotation process. Neji makes use of existing software tools and libraries for text processing, e.g., tokenization, sentence splitting, lemmatization, with some adjustments to meet the lexical specificities of biomedical texts. For concept recognition, Neji supports both dictionary-lookup matching and ML-based approaches by customizing existing libraries that implement these approaches. For instance, like BeCAS, it uses the CRF tagger implemented in Gimli. Hence, various CRF models trained for Gimli can be used in Neji, each model targeting a specific type of biomedical entities such as genes or proteins. Since Gimli does not perform disambiguation, Neji has introduced a simple algorithm to associate each recognized entity mention with a unique biomedical concept.

### Summary of benchmarking results

Tseytlin et al. [20] have conducted a comprehensive empirical study that includes five state-of-the-art semantic annotators that were compared based on the execution time and standard annotation performance metrics (precision, recall, F1-measure). Four of the benchmarked tools, namely cTAKES, MetaMap,^3^ ConceptMapper, and NOBLE Coder have been directly covered in the previous section, whereas the fifth tool - MGrep - was considered as a service used by NCBO Annotator in the first stage of its annotation process. The benchmarking was done on two publicly available, human-annotated corpora (see Table 5): one (ShARe) consisting of annotated clinical notes, the other (CRAFT) of annotated biomedical literature. Documents from the former corpus (ShARe) were annotated using the SNOMED-CT vocabulary (Table 1), while for the annotation of the latter corpus (CRAFT), a subset of OBO ontologies were used as recommended by the corpus developers.

The study showed that all the tools performed better on the clinical notes corpus (ShARe) than on the corpus of biomedical literature (CRAFT). The results demonstrated that on the ShARe corpus, NOBLE Coder, cTAKES, MGrep, and MetaMap were of comparable performance, while only ConceptMapper somewhat lagged behind. On the CRAFT corpus, NOBLE Coder, cTAKES, MetaMap, and ConceptMapper were quite aligned, whereas MGrep performed significantly worse, due to very low recall. In terms of speed, on both corpora, ConceptMapper proved to be the fastest one. It was followed by cTAKES, NOBLE Coder, and MGrep, respectively, whose speed was more-or-less comparable. However, MetaMap was by far the slowest (about 30 times slower than the best performing tool).

Another comprehensive empirical study that compared several semantic annotators with respect to their speed and the quality of the produced annotations is reported in [40]. The study included five contemporary annotators - Whatizit, MetaMap, Neji, Cocoa, and BANNER, which were compared on three manually annotated corpora of biomedical publications, namely NCBI Disease corpus, CRAFT, and AnEM (see Table 5). Evaluation on the CRAFT corpus considered 6 different biomedical entity types (e.g. species, cell, cellular component, gene and proteins), while on the other two corpora only the most generic type was considered, i.e., anatomical entity for AnEM, and disorder for NCBI. Two of the benchmarked annotators are either no longer available (Cocoa) or no longer maintained (BANNER^4^), whereas the other three were covered in the previous section. Benchmarking was done for each considered type of biomedical concept separately, and also using different configurations of the examined tools (e.g., five different term-to-concept matching techniques were examined).

The study showed that the tools’ performance varied considerably between various configuration options, in particular, various strategies for recognizing entity mentions in the input text. This variability in the performance associated with different configurations was also confirmed by Funk et al. [24]; we return to this topic in “Application-specific tool benchmarking” section.

Overall, Neji had the best results, especially on the CRAFT corpus, with significant improvements over the other tools on most of the examined concept types. Whatizit proved to have the most consistent performance across different configuration options, with an average variation of 4% in F1-measure. In terms of speed, Neji significantly outpaced the other tools.

## Entity-specific biomedical annotation tools

While the primary focus of this paper is on biomedical semantic annotation, and in particular general purpose biomedical semantic annotators, the work in the closely related area of biomedical Named Entity Recognition (NER) also deserves to be mentioned given that it has been a precursor to the biomedical semantic annotation technology. Early work in biomedical NER were mainly focused on developing dictionary-based, rule-based, or heuristics-based techniques for identifying entity mentions within chemical, biological, and medical corpora. Some of the earlier works include the work by Fukuda et al. [41] that used rules for extracting protein names, MedLEE [42] that employed contextual rules to perform mapping to an encoding table extracted from UMLS, and EDGAR [43] that extracted drugs and genes related to cancer. However, more advanced techniques based on machine learning (ML) models, more specifically Hidden Markov Models (HMM), Conditional Random Fields (CRF), and Support Vector Machines (SVM), have become more prominent in the recent years.

ABNER [44] was one of the earlier works that benefited from CRF models and was trained for five specific entity types, namely Protein, DNA, RNA, Cell Line, and Cell Type. ABNER extracted features based on regular expressions and n-grams to train a CRF model, and did not introduce any syntactic or semantic features in this process. Gimli [37] is a more recent NER toolkit that is also based on CRF models. The main advantage of Gimli is its introduction of a wide range of features, namely: orthographic, linguistic, morphological, external, and local context features. The orthographic features include capitalized mentions, counting, and symbol type features, while the linguistic features consist of word lemmas, POS tags, and products of dependency parsing. The morphological features cover n-grams and word shapes. The local and external features constitute gene and protein names as well as trigger words. The wide spectrum of features enables the CRF model to be highly accurate on different benchmark datasets including GENETAG and JNLPBA (see Table 5).

The work by Leaman et al. [45], known as DNorm, is a method specifically built for disease mention detection in biomedical text. DNorm is based on BANNER [46] for disease mention detection and subsequently uses a pairwise learning to rank framework to perform normalization. Similar in objective to DNorm but with focus on genes, SR4GN [47] is a rule-based system specifically built to link species with corresponding gene mentions. This tool has shown better performance compared to LINNAEUS [48], which is a tool for the same purpose built using a dictionary-based approach for mention detection and a set of heuristics for ambiguity resolution. The authors of SR4GN subsequently proposed GNormPlus that focuses on the identification of gene names and their identifiers. The distinguishing aspect of this tool is that it is able to distinguish gene, gene family, and protein domains by training a supervised CRF model on annotated gene corpora.

There have also been attempts at combining the benefits of rule-based methods and ML techniques. For instance, OrganismTagger [49] uses a set of grammar rules written in the JAPE language, a set of heuristics, as well as an SVM classifier to identify and normalize organism mentions in text including genus, species, and strains.

In a later publication [50], the developers of DNorm discussed the benefits of developing an entity type agnostic NER framework that could be retrained easily given sufficiently annotated training data and a related lexicon. Based on this objective, the TaggerOne tool was developed as an entity type independent tool that employs a semi-Markov structured linear classifier and has shown favorable performance on both NCBI Disease corpus as well as the chemical BioCreative 5 CDR corpus (see Table 5). In contrast to tools such as TaggerOne that rely only on a single ML model, there has also been work in the literature that rely on ensembles of models. For instance, tmChem, an ensemble built on BANNER and tmVar [51], focuses on the recognition of seven different types of chemical mentions in biomedical literature, namely Abbreviation, Family, Formula, Identifier, Multiple, Systematic and Trivial.

While the above approaches benefit from annotated corpora and some form of (semi) supervised training, such methods are task and entity dependent, and training them on new entity types is time consuming and resource intensive [52]. For this reason, unsupervised NER methods have started to emerge. For instance, the method proposed by Zhang and Elhadad [52] uses a noun chunker to detect possible entity candidates and subsequently categorizes the entity candidates based on distributional semantics. This method showed reasonable performance on the i2b2 and GENIA corpora (see Table 5).

It is worth mentioning that given the large amount of biomedical documents and texts that need to be processed by NER tools, several researchers have looked at optimizing the parallel capabilities of these tools. The work by Tang et al. [53] and Li et al. [54] are two notable recent work in this respect. These two works contend that given the sequential nature of CRF models, their parallelization is not trivial. On this basis, they show how the MapReduce framework can be used to efficiently train CRFs for biomedical NER.

It is also important to note that research and development of biomedical named entity recognition and normalization tools have been fostered through different initiatives of the biomedical research community. A notable one is the BioCreative initiative (http://www.biocreative.org/tasks/), a series of yearly challenges focused on text mining and information retrieval tasks relevant to the life science domain, including recognition of chemicals, genes, drugs, and diseases in biomedical texts. For instance, one of the tasks at the BioCreative IV challenge [55] was to automatically identify terms in a given article that refer to the concepts from the Gene Ontology (GO; see Table 1), that is, to semantically annotate articles with GO concepts. Benchmarking of the proposed solutions was done on the BC4GO corpus (see Table 5). The best performing team on this task applied a supervised classification method that relies on a knowledge base built by leveraging a large database of (over 100 K) MEDLINE abstracts annotated with GO terms [56]. In particular, the tool developed by this team, known as the GOCat tool, relies on similarities between an input text and already curated instances in the tool’s knowledge base, to annotate the input with the most prevalent GO terms among the instances from the knowledge base. The BioCreative V challenge hosted a Disease Named Entity Recognition (DNER) task [57], where the participating systems were given PubMed titles and abstracts and asked to return normalized disease concept identifiers. The benchmarking of the submitted solutions was done on the Chemical-Disease Relation (CRD) corpus (see Table 5). The best system (based on a CRF model with post-processing) achieved an F-score of 86.46%, a result that approaches the human inter-annotator agreement (0.8875). A large majority of the proposed solutions relied on ML (only 3 out of 16 were based exclusively on a dictionary-lookup method); one third of these solutions (4) used ML only, while others (8) exploited a combination of ML with dictionaries and/or pattern matching.

For a more comprehensive list of biomedical NER tools, in-depth discussion on the techniques, features and corpora used, the entity types that are covered and a comparative performance analysis, we refer the interested reader to the work by Campos et al. [58].

## Challenges

Even though significant efforts have been devoted to the development of sophisticated semantic annotation tools, there are still challenges that need to be resolved if these tools are to reach their full potential. This section points to some of those challenges, as well as to some of the existing research work that offers potential solutions.

### The lack of sufficient context for understanding entity mentions

An often cited source of difficulty associated with the recognition of entities in biomedical texts is the lack of sufficient context for interpreting the entity mentions [59]. For instance, Tseytlin et al. [20] reported that the largest proportion of annotation errors made by their NOBLE Coder annotator was due to the missing or incomplete context or background knowledge.

The collective annotation approach was proposed as a way of dealing with this challenge [59]. It relies on the global topical coherence of entities mentioned in a piece of text and is done by disambiguating a set of related mentions simultaneously. The basic idea is that if multiple entity mentions co-occur in the same sentence or paragraph, they can be considered semantically related. In particular, the approach proposed by Zheng et al. [59] consists of creating a document graph (*G*_*d*_) with entity mentions recognized in a document as nodes, while edges are established between those pairs of nodes (entity mentions) that co-occur in the same sentence or paragraph of the document. Each entity mention is then connected with one or more entity candidates from the knowledge base (KB) based on the name variants associated with entities in the KB. Finally, for each entity mention (*m*) - entity candidate (*c*) pair (*m,c*), a score is computed based on i) the general popularity of the candidate entity *c* in the KB, that is, its level of connectedness to other entities in the KB (non-collective score), and ii) level of connectedness of candidate *c* only with candidate concepts of entity mentions that are connected to mention *m* in the *G*_*d*_ graph. The candidate entity *c* from the (*m,c*) pair with the highest score is selected as the appropriate entity for the given entity mention *m*. A similar approach was proposed and proved effective for general purpose semantic annotators in work such as [60].

### Scaling to very large document sets

One of the weaknesses of today’s biomedical semantic annotators lies in their speed, that is, the time required for completing the annotation task on very large corpora (with tens and hundreds of millions of documents) [20]. Note that speed estimates given in Tables 3 and 4 (qualifying almost all examined tools as suitable for real-time processing) are based on the experimental results reported in the literature, where experiments were done with small corpora (up to 200 documents).

Divita et al. [61] aimed at using semantic annotation to improve information extraction and retrieval of clinical notes from the Veterans Informatics and Computing Infrastructure (VINCI) hosting huge and continuously growing amounts of medical notes. However, they found today’s annotators unapt for that task, as, based on the Divita et al., even when running on several multi-core machines, today’s annotators would need multiple years to index VINCI notes with semantic concepts. As a solution to this challenge, they proposed Sophia, an UMLS-based annotation tool, that deals with high throughput by replicating either certain components of the annotation pipeline or the entire pipeline [61]. Sophia is built from the components of the v3NLP framework [62], a suite of middleware text-processing components aimed for building various kinds of NLP applications. In particular, Sophia makes use of the v3NLP components for dealing with the idiosyncrasies of clinical texts, as well as the framework’s scaling-up and scaling-out functionalities for efficiently handling huge quantities of texts.

### Adaptation to new document type(s) and/or terminologies specific to particular biomedical subdomain

Another challenge originates in the variety of biomedical texts and differences among different kinds of text, particularly differences between biomedical literature and clinical text [20, 25]. According to Garla and Brandt [7], “clinical text is often composed of semi-grammatical **‘**telegraphic**’** phrases, uses a narrower vocabulary than biomedical literature, and is rife with domain-specific acronyms.” In addition, common to both clinical texts and scientific papers is the presence of local dialects, such as specific jargon developed within a medical center, or particular, idiosyncratic protein nomenclatures created within research laboratories [63]. Due to these issues, an annotation tool developed and/or configured for a particular type of medical texts or even one application case, tied to a particular medical institution/center, cannot be directly ported to some other text type and/or application case without, often significant, drop in performance.

A potential solution to this diversity in text types and terminologies is the use of flexible general-purpose annotation tools that can be configured to work with different text types and vocabularies [19]. In fact, Funk et al. [24] have demonstrated that if properly tuned, a generic annotation tool can offer better performance than tools designed specifically for particular biomedical task or domain. The key is in the modularity and flexibility of a tool so that one can precisely control how terms in the text are to be matched against the available terminologies.

While the majority of the annotators listed in Tables 3 and 4 were developed to be modular and flexible, their configuration is a complex task for users lacking expertise in NLP and not knowing the intricacies of the tool’s internal functioning. The latter is especially relevant as not all parameters equally affect the performance; also, the interaction of the parameters need to be considered.

Besides configuring the tool’s annotation method, e.g., kinds of text processing and term matching options, adaptation to a different biomedical (sub)domain also requires either development or, at least, customization of the dictionary that the tool uses to recognize concept mentions in the input text. While there are numerous ontologies, knowledge bases, thesauri, and similar kinds of biomedical resources that can be used for dictionary development, that task is often overly complex for regular users. This is because each tool has its own idiosyncratic structure and format for vocabulary representation and storage, designed to optimally match the tool’s annotation algorithm. To alleviate the task of dictionary development/customization, Tseytlin et al. [20] have developed an interactive terminology building tool, as a component of the NOBLE Coder annotator. The tool allows users to import existing terminologies (of various kinds), and then customize them by selecting only certain segments (branches) of the imported terminologies, and/or to filter them by semantic types. A tool of this type would be a useful complement to any semantic annotator that relies on a dictionary-lookup approach.

### Application-specific tool benchmarking

As argued in “Semantic Biomedical Annotation Tools” section, benchmarking of semantic annotators requires that each annotator is configured based on the specificities of the benchmarking task, so that it demonstrates its optimal performance on the task. The effect of configuration on the annotators’ performance was well demonstrated by Funk et al. [24] in their comprehensive empirical study that included MetaMap, ConceptMapper, and NCBO Annotator (see Semantic Biomedical Annotation Tools section). The researchers examined over 1000 parameter combinations in the context of the CRAFT evaluation corpus (Table 5) and 8 different terminologies (ontologies). They found that default parameter values often do not lead to the best performance, and that by appropriately setting parameters, F-measure can be significantly increased (even by 0.4 points). This suggests that if it is to be used for making a decision on the annotator to adopt in a particular application case, the benchmarking studies should not be based on the tools’ default configuration, but should include tools customized to the specific features of the application case.

Another requirement for application-specific benchmark study is the selection of appropriate evaluation corpora and terminology source for building or customizing the tools’ dictionaries. While numerous annotated corpora have been developed (Table 5), including both manually annotated gold standard corpora and corpora annotated in an automated or semi-automated way known as silver standards, the information about these resources are dispersed on the web and it takes time and effort to collect information about the available evaluation corpora and their features.

Considering the above stated difficulties associated with the setup of application-specific benchmarking studies, we point to the need for a benchmarking ‘toolkit’ that would facilitate the task of tool benchmarking in the context of a specific application case. An important component of such a toolkit would be a searchable registry of existing annotated corpora. For each corpus, the registry should include basic qualitative and quantitative information, e.g., the sources and types of documents that it includes, and the vocabularies or ontologies that were used for annotating the corpus, among others. In addition, the registry of annotated corpora would need to contain guidelines for how each corpus should be used, references to the studies where the corpus was previously used, and any additional information that might be of relevance for effective use of the given corpus.

Another important component of the benchmarking toolkit would be guidelines and/or tools for optimal configuration of annotation tools. The starting point for such guidelines could be the set of suggestions that Funk et al. [24] derived from their study, related to the selection of optimal parameter values based on the terminology (ontology) to be used for annotation. Tools enabling semi-automated or automated parameter tuning would greatly facilitate this task. Algorithmic procedures and tools developed for general purpose semantic annotators, like the one proposed in [64], could be adapted to tune parameters of biomedical annotators.

With such a benchmarking toolkit, it would be also possible to evaluate the performance of general purpose biomedical annotators on the tasks of recognizing and normalizing specific types of biomedical entities, e.g., chemicals, genes, drugs, or diseases. This would allow for evidence-based recommendation of appropriate semantic annotators for entity-specific tasks. While some initial work in this direction has been done by Campos et al. [40] (see Summary of benchmarking results section), only a small number of the current tools have been examined (some of the tools evaluated in their study are no longer available), and they were not tuned to the entity specific annotation tasks. Henceforth, new studies with current general purpose annotators, customized for the entity-specific task at hand, are needed in order to obtain conclusive evidence on the performance of the current tools for specific biomedical entity types.

### Semantic annotation in languages other than English

Large majority of tools, ontologies, and corpora developed for biomedical semantic annotation, and biomedical NLP in general, are for the English language. Semantic annotators discussed in the previous sections fall in this category of “English-only” tools. However, the development of NLP resources and tools for semantic annotation in languages other than English has started receiving increasing attention both in research and practice.

The CLEF (Conference and Labs of the Evaluation Forum) conference series have been hosting eHealth Labs where one of the tasks has been entity recognition and normalization, i.e., semantic annotation, in languages other than English, primarily French. Systems developed to face this challenge varied greatly [65, 66]. The team with the best performance at the latest eHealth Lab, held in conjunction with CLEF 2016, proposed a system that could be qualified as a general purpose semantic annotator [67]. In particular, to perform the entity recognition task, this system used Peregrine [68], a dictionary-based concept recognition tool, in conjunction with a dictionary consisting of French vocabularies from UMLS supplemented with automatically translated English UMLS terms. Several post-processing steps were implemented to reduce the number of false positives, such as filtering based on precision scores derived from the training data. Entity normalization relied on the <entity_mention, semantic_group, CUI^5^> combinations extracted from the training set.

Another important initiative was the CLEF-ER challenge that took place in 2013 as part of the Mantra project aimed at providing multilingual documents and terminologies for the biomedical domain [69]. For this challenge, Medline and biomedical patent documents were released in five languages: English, German, French, Spanish, and Dutch. Mappings to English documents were provided for all documents that were in a language other than English, though the mappings were not available between all pairs of languages, e.g., between Spanish and German. The organizers also released the CLEF-ER terminology, a multilingual vocabulary with term synonyms in the above mentioned five languages.^6^ The challenge received several submissions dealing with various challenges of multilingual biomedical NLP, including semantic annotation, e.g. [70, 71], and the creation of multilingual corpora, e.g., [72, 73]. An interesting approach to multilingual semantic annotation was proposed by Attardi et al. [71]. The method starts from the English language Silver Standard Corpus (SSC) provided by the CLEF-ER organizers [72], which is first translated into a target language corpus, and then entity annotations are ‘transferred’ to it. The translation is done using an open-source toolkit for statistical phrase-based machine translation. The word alignment information produced by the translation tool is used to determine the correspondence between entities in the source and the target language sentences. The resulting annotated corpus is referred to as the Bronze Standard Corpus (BSC). In addition, a dictionary of entities is also created, which associate each <entity_mention, semantic_group > pair with all the corresponding CUIs that appeared in the SSC. The BSC is used to train a Named Entity detection model, which is aimed at associating entity mentions with their semantic groups. This model is then used for tagging entity mentions in the target language sentences with the proper semantic group. Finally, after entity mentions have been assigned to their semantic group, each mention is linked to corresponding CUIs by looking up CUIs associated with the <entity_mention, semantic_group > pairs in the previously built dictionary.

## Conclusions

In this paper we have analyzed the current state of the art in the domain of general purpose biomedical semantic annotators, and pointed to some of the areas where further research and development is needed to improve the performance of the current solutions and make them robust to the requirements of real-world biomedical applications. In conclusion, we can say that the majority of the analyzed tools proved to be highly modular and configurable, thus fulfilling the promise of general purpose biomedical annotators as annotators adaptable to different areas of biomedicine. In addition, the majority of the examined tools are made publicly available as open-source software libraries, thus bootstrapping further developments in biomedical semantic annotation. As areas that require further research and development, we have identified: i) finding new, more effective ways of dealing with the often terse context of biomedical entity mentions, especially in clinical texts; ii) improving the scalability of annotators so that they can efficiently process biomedical corpora with tens and hundreds of millions of documents; iii) development of auxiliary tools that would facilitate the task of customizing an annotator to the requirements of a particular annotation task, iv) development of a toolkit for benchmarking semantic annotators in the context of a specific application case, and thus enabling users to make well-informed decisions regarding the annotator to use in their particular application setting, and vi) continuing and intensifying research efforts aimed at multilingual biomedical semantic annotation.

We have also pointed to some of the potential benefits and application cases of biomedical semantic annotation technology in order to demonstrate and exemplify the opportunities that this technology can bring about, and thus encourage the research community to put efforts in overcoming the identified challenges and bring the tools to their full potential. We believe that there is a tremendous potential in using biomedical semantic annotation technology for processing, analyzing and structuring unstructured textual biomedical content both in the form of clinical and research material, and hope that this review paper provides the means to encourage the community to further investigate and adopt this technology.

## Abbreviations

CRF: Conditional random fields
CUI: Concept unique identifier
KB: Knowledge base
ML: Machine learning
NER: Named entity recognition
NLP: Natural language processing

## Footnotes

1 At the time of writing this manuscript, the given list of tools could be considered exhaustive with respect to the given selection criteria, i.e., we included all the tools that met the given set of criteria and were reported in the literature. However, considering the pace of new developments, it is reasonable to expect new tools with the given characteristics soon to emerge.

2 Speed is characterized only from the perspective of the tool’s usability for real-time text annotation; we do not report exact operation time since it can vary considerably depending on the tool’s configuration, the characteristics of the corpora, the machine the tool is running on.

3 The study used MMTX (https://mmtx.nlm.nih.gov/), Java implementation of MetaMap, which produces only slightly different results than MetaMap [20] due to differences in tokenization and lexicalization procedures.

4 Source code is available from http://banner.sourceforge.net/ but the last update was in year 2011.

5 CUI stands for Concept Unique Identifier, that is, a unique identifier of a concept in a knowledge base, such as UMLS Metathesaurus.

6 This terminology can be accessed from the project output page of the Mantra project website: https://sites.google.com/site/mantraeu/project-output
